# Evolution, gene expression, and protein‒protein interaction analyses identify candidate CBL-CIPK signalling networks implicated in stress responses to cold and bacterial infection in citrus

**DOI:** 10.1186/s12870-022-03809-0

**Published:** 2022-09-01

**Authors:** Cui Xiao, Hu Zhang, Fan Xie, Zhi-Yong Pan, Wen-Ming Qiu, Zhu Tong, Ze-Qiong Wang, Xiu-Juan He, Yu-Hai Xu, Zhong-Hai Sun

**Affiliations:** 1 Fruit and Tea Research Institute, Hubei Academy of Agricultural Sciences, Wuhan, 430064 China; 2 Key Laboratory of Horticultural Plant Biology (Ministry of Education), Huazhong Agricultural University, Wuhan, 430070 China

**Keywords:** Citrus, CBL-CIPK interaction, Evolution, Expression pattern, Abiotic and biotic stresses

## Abstract

**Background:**

Cold is a major abiotic stress and Huanglongbing and citrus canker disease are two devastating bacterial diseases for citrus. The Ca^2+^-CBL-CIPK network is known to regulate different types of stress signalling in plants. How do CBL–CIPK signalling networks function in response to cold and infection by *C*Las or *Xcc* in citrus?

**Results:**

Eight calcineurin B-like proteins (CBLs) and seventeen CBL-interacting protein kinases (CIPKs) were identified from the cold-tolerant satsuma mandarin ‘Guijing2501’ (*Citrus. unshiu*) and *C*Las/*Xcc*-sensitive sweet orange (*C. sinensis*). Phylogenetic analysis revealed that both CBL and CIPK family members in citrus were classified into an ancient and a recent clade according to their conserved domain characteristics and/or intron/exon structures. Genome duplication analysis suggested that both tandem and segmental duplications contributed to the amplification of the *CBL* and *CIPK* gene families in citrus under intense purifying selection, and the duplication events only existed in the recent clades. Expression comparison of the duplicated gene pairs indicated that the duplicated *CBL* and *CIPK* genes underwent functional differentiation. Further expression analysis identified that *CBL1*, *5*, *6*, and *8* and *CIPK2*, *8*, *12*, *15*, *16*, and *17* were significantly regulated by multiple stresses, including cold, *Xcc* infection and/or *C*Las infection, in citrus, whereas *CBL2*/*7* and *CIPK1/4/5/11*/*13*/*14* were independently highly regulated by cold and *CIPK3* was uniquely responsive to *Xcc* infection. The combination analyses of targeted Y2H assay and expression analysis revealed that CBL6-CIPK8 was the common signalling network in response to cold and *Xcc* infection, while CBL6/CBL8-CIPK14 was uniquely responsive to cold in citrus. Further stable transformation and cold tolerance assay indicated that overexpression of *CuCIPK16* enhanced the cold tolerance of transgenic *Arabidopsis* with higher POD activity and lower MDA content.

**Conclusions:**

In this study, evolution, gene expression and protein‒protein interaction analyses of citrus CBLs and CIPKs were comprehensively conducted over a genome-wide range. The results will facilitate future functional characterization of individual citrus *CBLs* and *CIPKs* under specific stresses and provide clues for the clarification of cold tolerance and disease susceptibility mechanisms in corresponding citrus cultivars.

**Supplementary Information:**

The online version contains supplementary material available at 10.1186/s12870-022-03809-0.

## Background

Specific mechanisms have evolved in plants to detect and respond to various abiotic and biotic stresses. Calcium plays an essential role in sensing and signalling these stresses in plants. Under specific stress conditions, plants can release stress-induced Ca^2+^ signals, which are subsequently decoded by various Ca^2+^-sensors [1]. Calmodulin (CaM), calmodulin-like protein (CML), calcineurin B-like protein (CBL) and calcium-dependent protein kinase (CDPK) are four sensors of Ca^2+^ [2]. CDPK, which has a kinase domain, can function independently of protein kinases [3]. However, CaM, CML and CBL harbour no kinase domain and require interactions with specific protein kinases to activate phosphorylation cascades and regulate downstream gene expression [4, 5].

Complexes of CBL and CBL-interacting protein kinase (CIPK) have been identified as calcium-decoding signalling networks that decode intracellular calcium signals and mediate the response of downstream genes to various stresses [1]. With a highly conserved core region that is composed of four elongation factor hand (EF-hand) domains, CBLs are responsible for Ca^2+^ binding and interaction with CIPKs [1, 5, 6]. Furthermore, the PFPF motif located at the C-terminus is critical for CBL phosphorylation by CIPKs [1]. CIPK proteins are classified into the sucrose nonfermenting 1-related serine/threonine kinases 3 (SnRK3) subfamily [7] and consist of an N-terminal catalytic kinase domain and a self-inhibitory C-terminal regulatory domain [8]. The N-terminal catalytic kinase domain contains an ATP binding site and an activation loop, and the C-terminal regulatory domain harbours a unique NAF/FISL motif and a protein-phosphatase interaction (PPI) motif [1]. Ca^2+^-bound CBLs can interact with the conserved NAF/FISL motif and activate the catalytic activity of target CIPKs [9]. Some CIPKs can also target specific members of the protein phosphatase 2C (PP2C) by the PPI motif [10].

In previous studies, the CBL–CIPK signalling networks have been extensively investigated in response to nutrient deficiency and salt stress in plants. For instance, AtCBL1/9-AtCIPK23 and AtCBL4-AtCIPK6 complexes can phosphorylate AKT1 and AKT2, respectively, and then modulate their activities to maintain K^+^ homeostasis under low-K^+^ stress in *Arabidopsis* [11–13]. In addition, *AtCIPK24*, a well-known gene identified in the salt overly sensitive (SOS) pathway of *Arabidopsis*, can interact with *AtCBL4* and *AtCBL10* [14, 15]. The AtCBL4-AtCIPK24 complex is responsive to salt stress and controls long-distance Na^+^ transport from the root to the shoot, while the AtCBL10-AtCIPK24 complex functions mainly in the response of shoots to salt toxicity [14, 15]. However, there have been few studies of the CBL–CIPK network in response to cold stress [1]. To the best of our knowledge, the AtCBL1-AtCIPK7 complex may play a role in the cold response of *Arabidopsis* [16]. In addition, the CBL–CIPK network also plays important functions in response to pathogen elicitors [1]. For example, the OsCBL2-CIPK31-AKT1L, TaCBL4-TaCIPK5 and MaCBL1/9-MaCIPK23 complexes can positively regulate disease resistance in rice, wheat and cassava, respectively [17–19]. Overall, these findings suggest that CBL–CIPK signalling networks may have important functions in response to abiotic and biotic stresses in plants.

Citrus is one of the most widely cultivated fruit crops in the world. Cold is a major limiting factor affecting citrus production and survival in citrus northern fringe growing areas [20–22]. ‘Guijing2501’ satsuma mandarin (*Citrus. unshiu*) is a novel citrus cultivar with cold tolerance selected from Hubei Province in China, which is a northern fringe region for citrus growing [23]. Citrus Huanglongbing (HLB) disease, mainly caused by *Candidatus* Liberibacter asiaticus (*C*Las), and citrus canker disease, caused by *Xanthomonas citri* subsp. *citri* (*Xcc*), are two devastating bacterial diseases that affect the global citrus industry [24–27]. Sweet orange (*C. sinensis*) is a citrus cultivar sensitive to *C*Las and *Xcc* infection. CBL-CIPK interactions are known to regulate different types of stress signalling in plants. Although genome-wide analysis has identified *CBL* and *CIPK* family genes as involved in various stresses in many fruit crops, including grape [28], apple [29], pineapple [30] and pear [31], and coexpression of *VvCBL* and *VvCIPK* combined with *Arabidopsis* CBL-CIPK interaction networks retrieved from STRING was employed to predict VvCBL-VvCIPK interactions in grape [28], few genome-wide studies in fruit crops have verified the CBL–CIPK interaction networks by protein‒protein interaction experiments. To the best of our knowledge, MdCIPK24-LIKE1 can physically interact with MdCBL1/4/10 to increase salt tolerance in apple [32]. In a previous study, citrus *CBL* and *CIPK* genes were identified in response to drought and arbuscular mycorrhizal fungi colonization according to expression data [33]. How do CBL–CIPK signalling networks function in response to cold and infection by *C*Las or *Xcc* in citrus? In this study, evolution, gene expression and protein‒protein interaction analyses were comprehensively conducted over a genome-wide range to identify candidate CBL-CIPK networks implicated in regulating responses to cold in ‘Guijing2501’ and *C*Las and *Xcc* infection in sweet orange. The findings may facilitate future functional characterization of individual citrus *CBLs* and *CIPKs* under specific stresses and provide clues for the clarification of cold tolerance and disease susceptibility mechanisms in corresponding citrus cultivars.

## Results

### Identification and cloning of *CBLs* and *CIPKs* in citrus

**Fig. 1:**
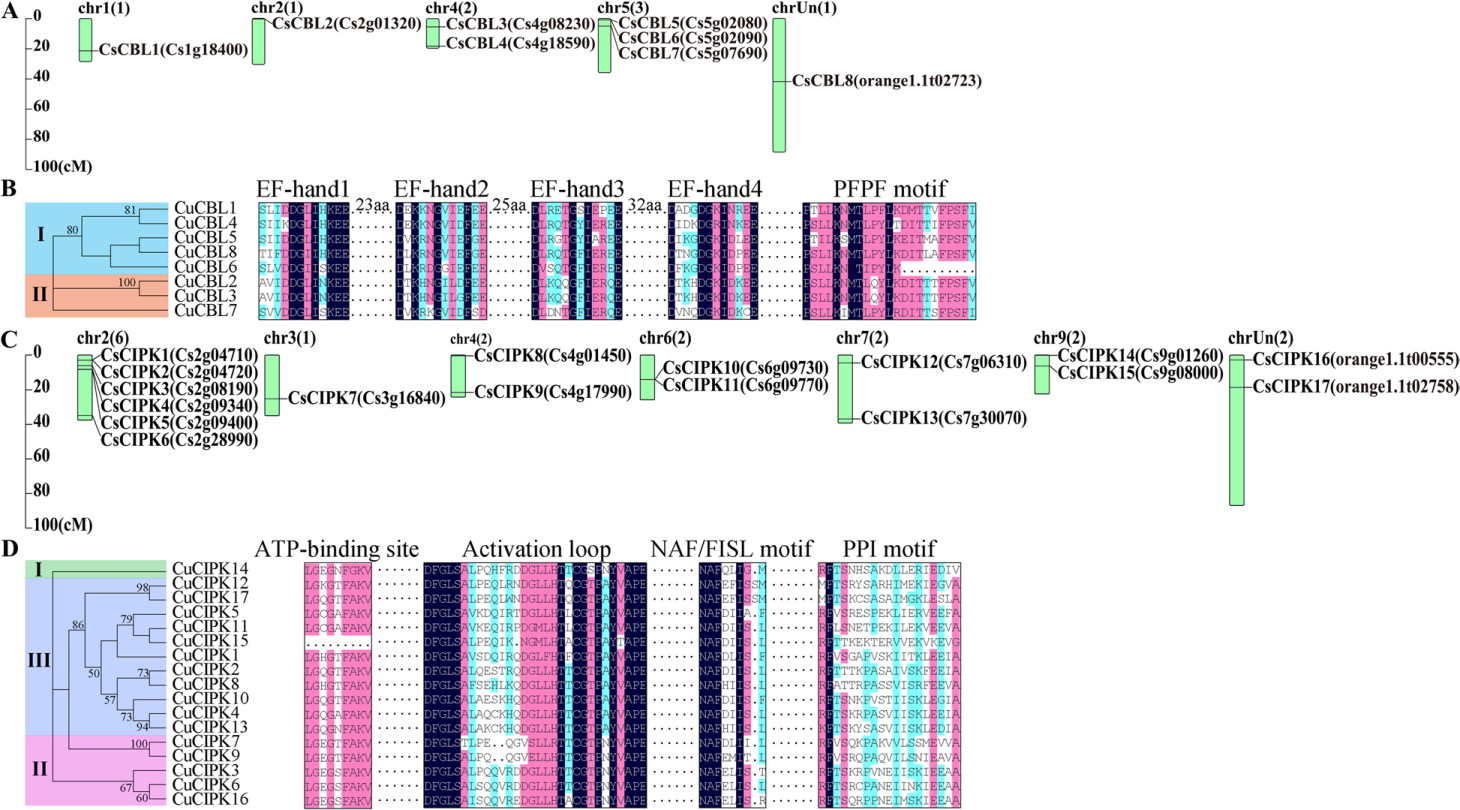
Chromosomal locations of *CsCBLs* (**A**) and *CsCIPKs* (**C**) and the phylogenetic trees based on the predicted CuCBL and CuCIPK protein sequences along with their sequence alignment in the conserved domains (**B** and **D**). Note that only chromosomes with *CsCBL* and *CsCIPK* genes are shown in the drawing. The phylogenetic trees for CuCBLs and CuCIPKs of ‘Guijing2501’ satsuma mandarin were generated using the maximum likelihood method. Four EF-hand domains, PFPF motifs and the lengths of the linker amino acid sequences for CuCBL proteins are shown in **B**. The ATP-binding site and activation loop in the N-terminal catalytic kinase domain and the NAF/FISL and PPI motifs in the C-terminal regulatory domain for CuCIPK proteins are shown in **D**

A total of eight *C. sinensis CBL* (*CsCBL*) candidate genes and 17 *CsCIPK* candidate genes were identified from the sweet orange genome, and they were named *CsCBL1*–*8* and *CsCIPK1*–*17* based on their positions in the chromosomes. These *CsCBL* and *CsCIPK* genes exhibited uneven distributions on chromosomes (Fig. 1A and 1C). We further analysed the intron/exon structures of these genes using the gene sequences (Table S1). As shown in Figure S1, all *CsCBL* genes were intron rich, whereas the *CsCIPK* genes could be divided into intron-rich and intron-less clades.

In addition, eight *C. unshiu CBL* (*CuCBL*) and 17 *CuCIPK* genes with full-length CDSs were cloned from the cold-tolerant ‘Guijing2501’ satsuma mandarin. Multiple sequence alignment analysis revealed that the deduced proteins of all the *CuCBL* genes from ‘Guijing2501’ contain four EF-hand domains and a PFPF motif. The EF1 and EF2, EF2 and EF3, and EF3 and EF4 domains were separated by 23, 25 and 32 amino acids, respectively, for all CuCBL proteins (Fig. 1B, File S1). All CuCIPK proteins except CuCIPK15 contain an ATP-binding site and an activation loop in the N-terminal catalytic kinase domain and an NAF/FISL motif and a PPI motif in the C-terminal regulatory domain (Fig. 1D, File S2).

### Phylogenetic analysis of *CBL* and *CIPK* family genes

**Fig. 2:**
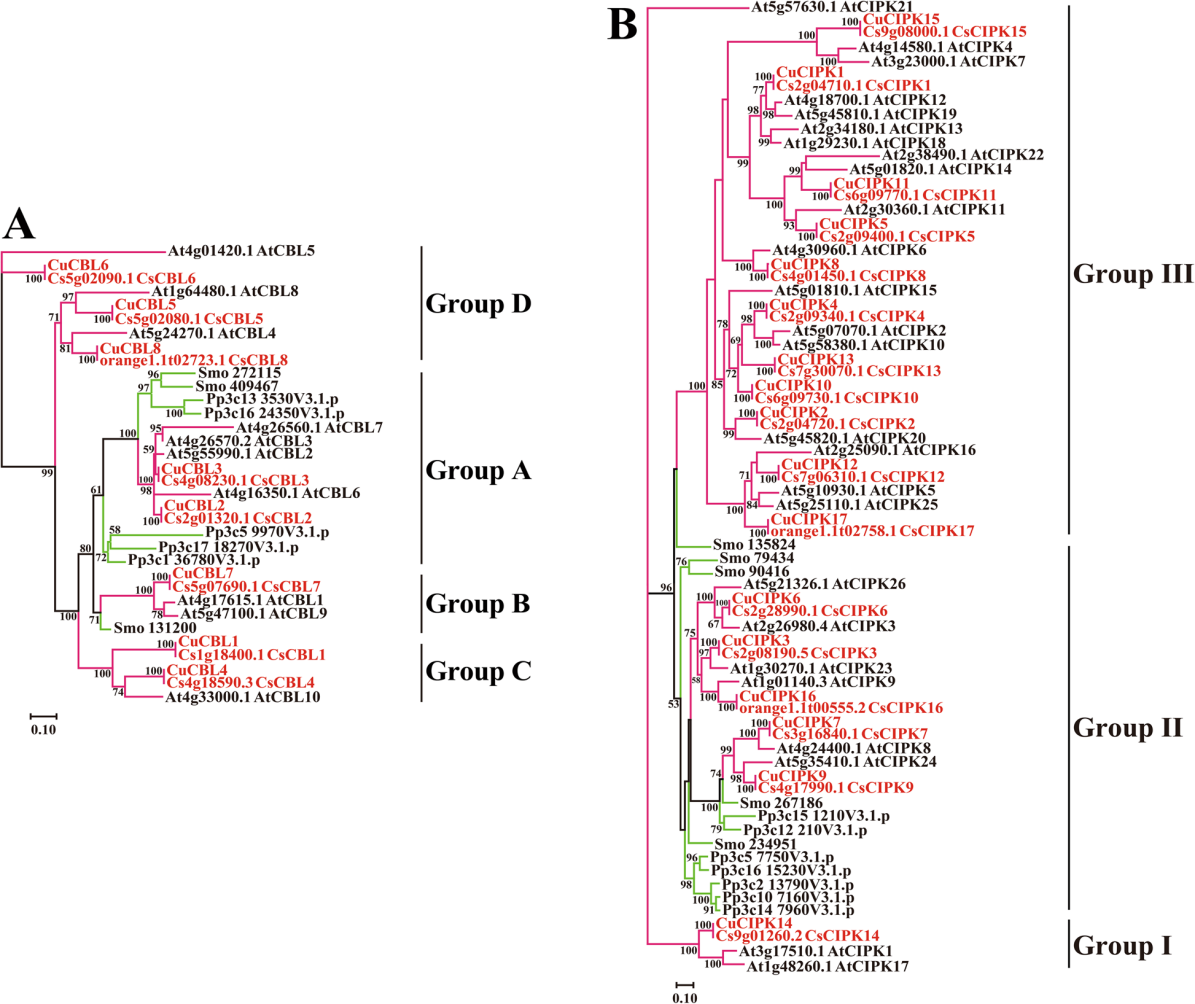
Phylogenetic tree of CBL (**A**) and CIPK (**B**) proteins. Eight Cs (u) CBLs and 17 Cs (u)CIPKs from sweet orange and ‘Guijing2501’ satsuma mandarin, 10 AtCBLs and 26 AtCIPKs from *Arabidopsis*, five CBLs and seven CIPKs from *Physcomitrella patens*, and three CBLs and five CIPKs from *Selaginella moellendorffii* were used to generate the maximum likelihood tree by using MEGA 7.0 with 1000 bootstraps. Cs (u) CBLs and Cs (u) CIPKs from citrus are highlighted in red. Branch lines of CBLs and CIPKs from eudicots are marked with rose red, and those of CBLs and CIPKs from ancient *bryophyta* and *pteridophyta* are marked with light green

To better understand the evolutionary relationship of the CBL and CIPK members, a maximum likelihood phylogenetic tree was generated based on the full deduced amino acid sequences of the CBL and CIPK family proteins from sweet orange, ‘Guijing2501’ satsuma mandarin, *Arabidopsis* and two ancient species (*Physcomitrella patens* and *Selaginella moellendorffii*) (Fig. 2, Tables S2-S5). Based on the phylogenetic tree, both the predicted citrus CBL and CIPK proteins could be divided into an ancient clade and a recent clade (Fig. 2). Cs(u)CBL2, 3 in Group A and Cs(u)CBL7 in Group B might be ancient genes because they were clustered with the CBLs from ancient species. However, Cs(u)CBL1, 4 in Group C and Cs(u)CBL5, 6, 8 in Group D might have evolved recently, as both groups included no members from the ancient species. Similarly, Cs(u)CIPKs in Group I and Group II might have evolved long ago; in contrast, the remaining Cs(u)CIPKs in Group III might have evolved recently. More interestingly, CIPKs in the ancient clade are intron rich, whereas CIPKs in the recent clade are intron less (Figure S1).

### Expression analysis of *CuCBL* and *CuCIPK* genes in cold-tolerant ‘Guijing2501’ satsuma mandarin under cold stress

**Fig. 3:**
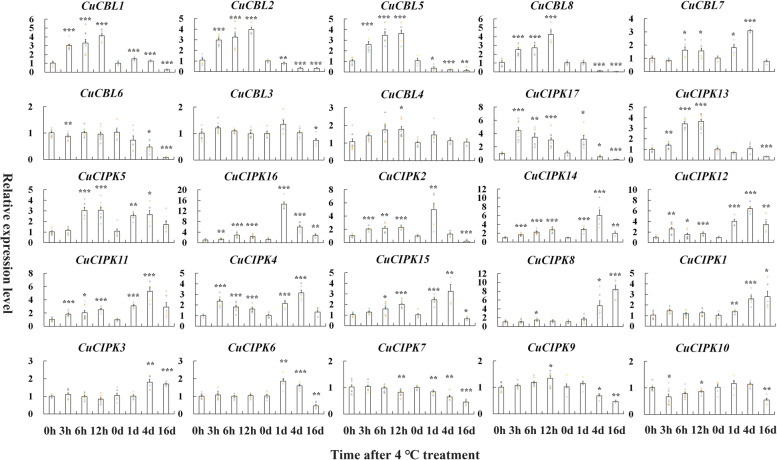
Expression patterns of eight *CuCBLs* and 17 *CuCIPKs* in cold-tolerant ‘Guijing2501’ satsuma mandarin under cold stress as determined by qRT‒PCR. Two-year-old healthy and uniform bud-grafting ‘Gujing2501’ satsuma mandarin trees growing in a greenhouse were subjected to low-temperature (4 °C) treatment as described in the Methods section. Three biological replicates were used for measuring gene expression, and the experiment was repeated once with similar results. The relative expression levels of candidate genes were calculated using the 2^−∆∆CT^ method. The *CuEF1α* gene was used as an internal reference gene for normalization of the transcript levels of the tested genes. Error bars indicate inferential statistics according to the SE. *indicates statistical significance (*p < 0.05; **p < 0.01; ***p < 0.001; Student’s t test) when compared to the expression level at 0 h/d

To test whether the *CBL* and *CIPK* family genes are responsive to cold stress in citrus,‘Gujing2501’ trees were subjected to low temperature (4 °C). Leaf samples were collected from the tree under test at 0, 3, 6, and 12 h and 0, 1, 4, and 16 days after the cold treatment. The gene expression levels of all the *CuCBL* and *CuCIPK* genes were determined by qRT‒PCR (Fig. 3, Table S6). The results showed that 75% of *CuCBL* genes (except *CuCBL3*, *4*) exhibited significant transcriptional changes upon cold treatment. *CuCBL1*, *2*, *5*, and *8* genes were upregulated 2.4 ~ 4.2-fold at the early time points (3 ~ 12 h). Almost all *CuCBL* genes were downregulated at the later time points (4 ~ 16 days), except that *CuCBL7* was upregulated 3.1-fold at 4 days. In particular, the recently evolved *CuCBL5*, *6*, and *8* genes were downregulated by 12.5 ~ 50.0-fold at 16 days after cold stress. In the case of *CuCIPK* genes, almost all *CuCIPK* genes were significantly upregulated upon cold treatment, except that *CuCIPK3*, *6*, *7*, *9*, and *10* remained at a relatively stable expression level. Among the upregulated *CuCIPK* genes*,* only *CuCIPK8* and *CuCIPK1* were upregulated at the later time points (4 ~ 16 days), and reached the peak expression level at 16 days. The remaining *CuCIPK* genes were upregulated from early time points. *CuCIPK17* reached the peak of expression level at 3 h, *CuCIPK13* and *5* at 12 h, *CuCIPK16* and *2* at 1 day, and *CuCIPK14*, *12*, *11*, *4*, and *15* at 4 days. Thus, these genes exhibited significant cold-induced transcriptional changes, implying that they may be functionally involved in mounting the physiological response of ‘Guijing2501’ satsuma mandarin to cold stress.

### Expression analysis of *CsCBL* and *CsCIPK* genes during *C*Las or *Xcc* infection in sweet orange

**Fig. 4:**
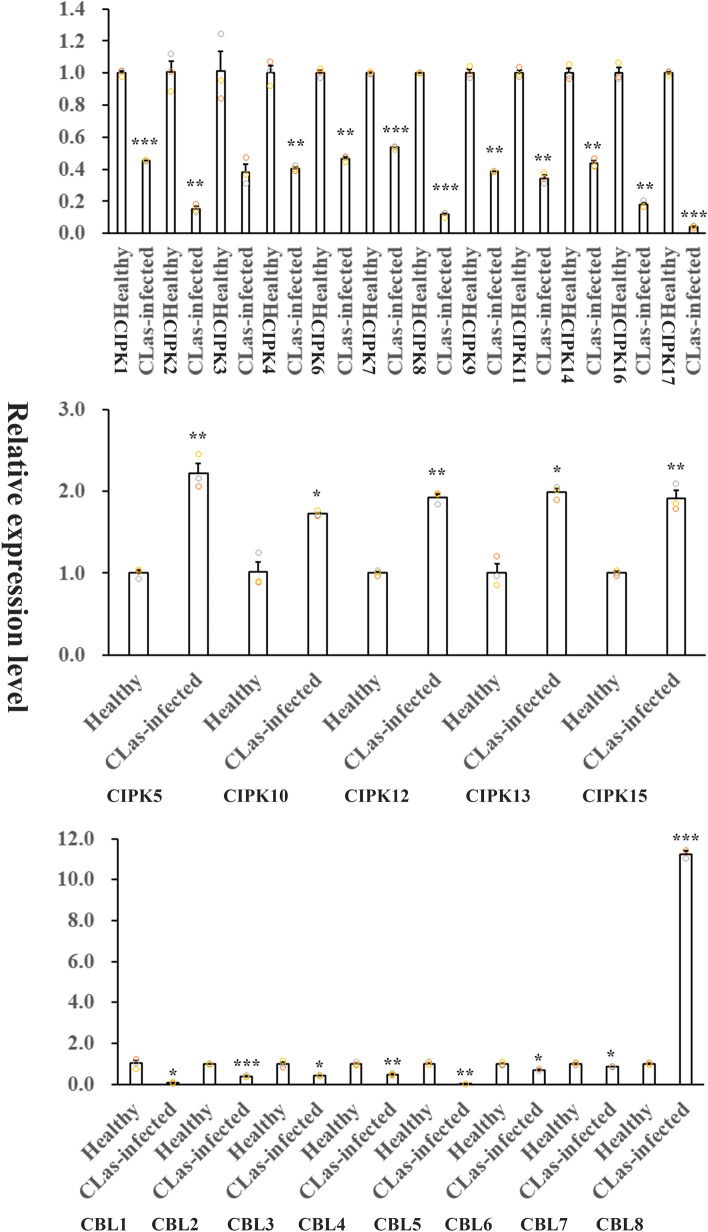
Expression patterns of eight *CsCBLs* and 17 *CsCIPKs* in leaves of healthy sweet orange trees and in leaves of those infected with *C*Las as determined by qRT‒PCR. Healthy and *C*Las-infected leaves from 15-year-old ‘Yuanfeng’ navel orange trees were collected in the same orchard in Wugang County of Hunan Province. Three biological replicates were used for measuring gene expression, and the experiment was repeated once with similar results. The relative expression levels of candidate genes were calculated using the 2^−∆∆CT^ method. The *CsEF1α* gene was used as an internal reference for the nominalization of the transcript levels of the genes tested. Error bars indicate inferential statistics according to the SE. *indicates statistical significance (*p < 0.05; **p < 0.01; ***p < 0.001; Student’s t test) when compared to the expression level in leaves of healthy sweet orange trees

**Fig. 5:**
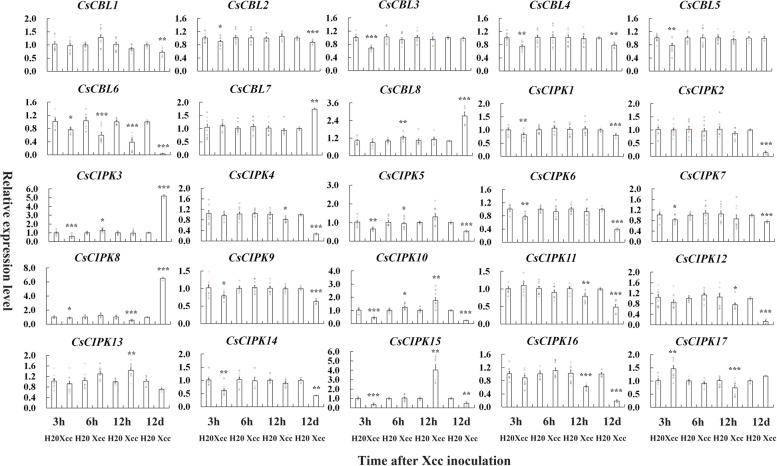
Expression patterns of eight *CsCBLs* and 17 *CsCIPKs* in leaves of sweet orange trees infiltrated by water and in leaves of those infiltrated by *Xcc *bacteria as determined by qRT‒PCR. Two-year-old healthy and uniform bud-grafting ‘Taoye’ sweet orange trees growing in a greenhouse were subjected to the *Xcc* inoculation assay as described in the Methods section. Three biological replicates were used for measuring gene expression, and the experiment was repeated once with similar results. The relative expression levels of candidate genes were calculated using the 2^−∆∆CT^ method. The *CsEF1α* gene was used as an internal reference for the nominalization of the transcript levels of the genes tested. Error bars indicate inferential statistics according to the SE. *indicates statistical significance (*p < 0.05; **p < 0.01; ***p < 0.001; Student’s t test) when compared to the expression level in leaves of sweet orange trees infiltrated by water

To determine whether the *CBL* and *CIPK* family genes are responsive to *C*Las and *Xcc* infection in citrus, the expression patterns of all *CsCBL* and *CsCIPK* genes from *C*Las-infected orange leaves (verified by HLB typical symptoms and *C*Las titre quantification; Figure S2A and Figure S2B) and *Xcc*-inoculated orange leaves (Figure S2C) were evaluated by qRT‒PCR analysis (Fig. 4–5; Table S7 and Table S8). The results showed that only a few *CsCBL* and *CsCIPK* genes exhibited significant transcriptional changes during *C*Las and *Xcc* infection. For example, the expression of *CsCBL8* showed a significant increase in *C*Las-infected trees (11.2 x) and in trees after 12 days of *Xcc* inoculation (2.7 x). In contrast, the expression of *CsCBL1* and *CsCBL5* was reduced by 14.3 × and 33.3 x, respectively, in *C*Las-infected trees, and the expression of *CsCBL6* was reduced by 33.3 × in trees after 12 days of *Xcc* inoculation. These expression patterns suggest that the recently evolved *CsCBL1*, *5*, *6* and *8* genes might also play important roles in the response to bacterial infection*.* Among the *CsCIPK* genes, the expression of *CsCIPK2*, *8*, *16* and *17* was decreased 5.6–25.0-fold in the *C*Las-infected trees; that of *CsCIPK2*, *12* and *16* was reduced 5.6–7.7-fold in trees after 12 days of *Xcc* inoculation, while that of *CsCIPK3* and *8* was increased 5.2- and 6.5-fold in trees after 12 days of *Xcc* inoculation, respectively. Only one gene, *CsCIPK15*, was upregulated (4.0-fold) at the early time points (12 h) after *Xcc* inoculation. The above expression data suggest that these seven genes (*CsCIPK2*, *3*, *8*, *12*, *15*, *16*, *17*) might be implicated in the responses of sweet orange to bacterial infection.

### Synteny analysis of *CsCBLs* or *CsCIPKs* within the sweet orange genome

**Fig. 6:**
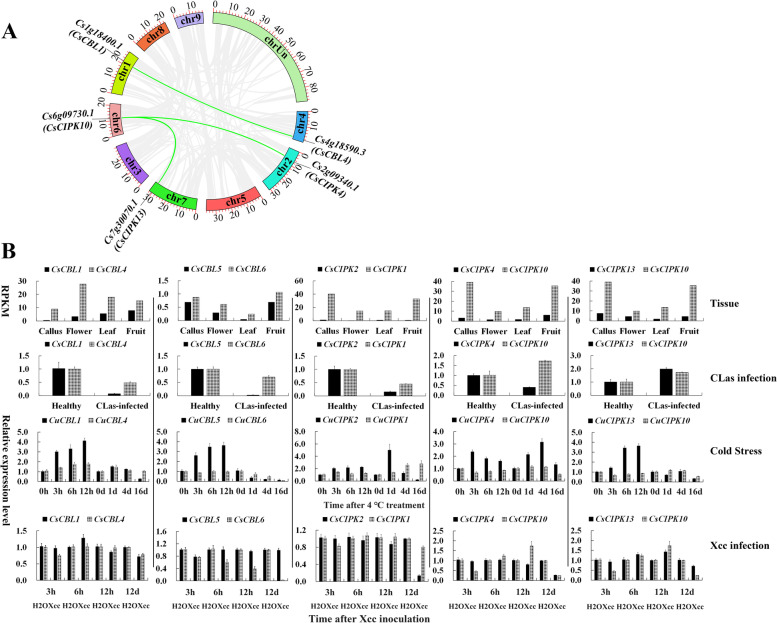
Segmental duplication gene pairs of *CsCBLs* and *CsCIPKs* within the sweet orange genome (**A**) and divergent expression patterns of the duplication gene pairs in tissues and under different abiotic and biotic stresses (**B)**. A. Grey lines in the background show the collinear blocks within the sweet orange genome, and the segmental duplication gene pairs of *CsCBLs* and *CsCIPKs* are highlighted by light green lines. B. The expression patterns in different tissues were analysed based on the RPKM values of RNA-seq data from the sweet orange genome database [34]

Duplication and diversification play essential roles in the evolution and expansion of gene families. One Step MCScanX was conducted within the sweet orange genome by TBtools software to identify the duplication events for the *CsCBL* and *CsCIPK* gene families. The results revealed that *CsCBL5/CsCBL6* and *CsCIPK1/CsCIPK2* were tandemly duplicated gene pairs, while *CsCBL1*/*CsCBL4*, *CsCIPK4*/*CsCIPK10* and *CsCIPK10*/*CsCIPK13* were segmentally duplicated gene pairs (Fig. 6A, Table S9). Interestingly, all the above duplicated genes were classified into recently evolved clades. In addition, the Ka/Ks ratios for all the duplicated gene pairs were lower than 0.5, suggesting that the *CsCBL* and *CsCIPK* gene families have evolved under intense purifying selection (Table S9).

In addition, subfunctionalization and/or neofunctionalization are major evolutionary fates of duplicated genes, in which a large proportion of the duplicated genes undergo functional differentiation through gene expression divergence [35]. In this study, except for *CsCBL5*/*CsCBL6*, the other four duplicated genes shared the same patterns: one was responsible for normal tissue development, and the other was in response to abiotic and/or biotic stress (Fig. 6B). For example, *CsCBL4*, *CsCIPK1* and *CsCIPK10* were highly expressed in tissues including callus, flower, leaf and fruit, while the corresponding duplicated genes *CsCBL1*, *CsCIPK2* and *CsCIPK4*/*13* were significantly upregulated by cold stress and downregulated by *C*Las infection and/or *Xcc* infection. Although both *CsCBL5* and *CsCBL6* were expressed at low levels in tissues, *CsCBL5* was significantly upregulated by cold stress and downregulated by *C*Las infection, whereas *CsCBL6* was significantly downregulated by cold stress and *Xcc* infection. These results suggest that duplication might play an important role in the expansion of the *CsCBL* and *CsCIPK* gene family, which might also contribute to the adaptive feature of citrus *CBL* and *CIPK* for cold and bacterial infection.

### Interactions and expression patterns of *CBLs* and *CIPKs* in the stress response to cold and bacterial infection in citrus

**Fig. 7:**
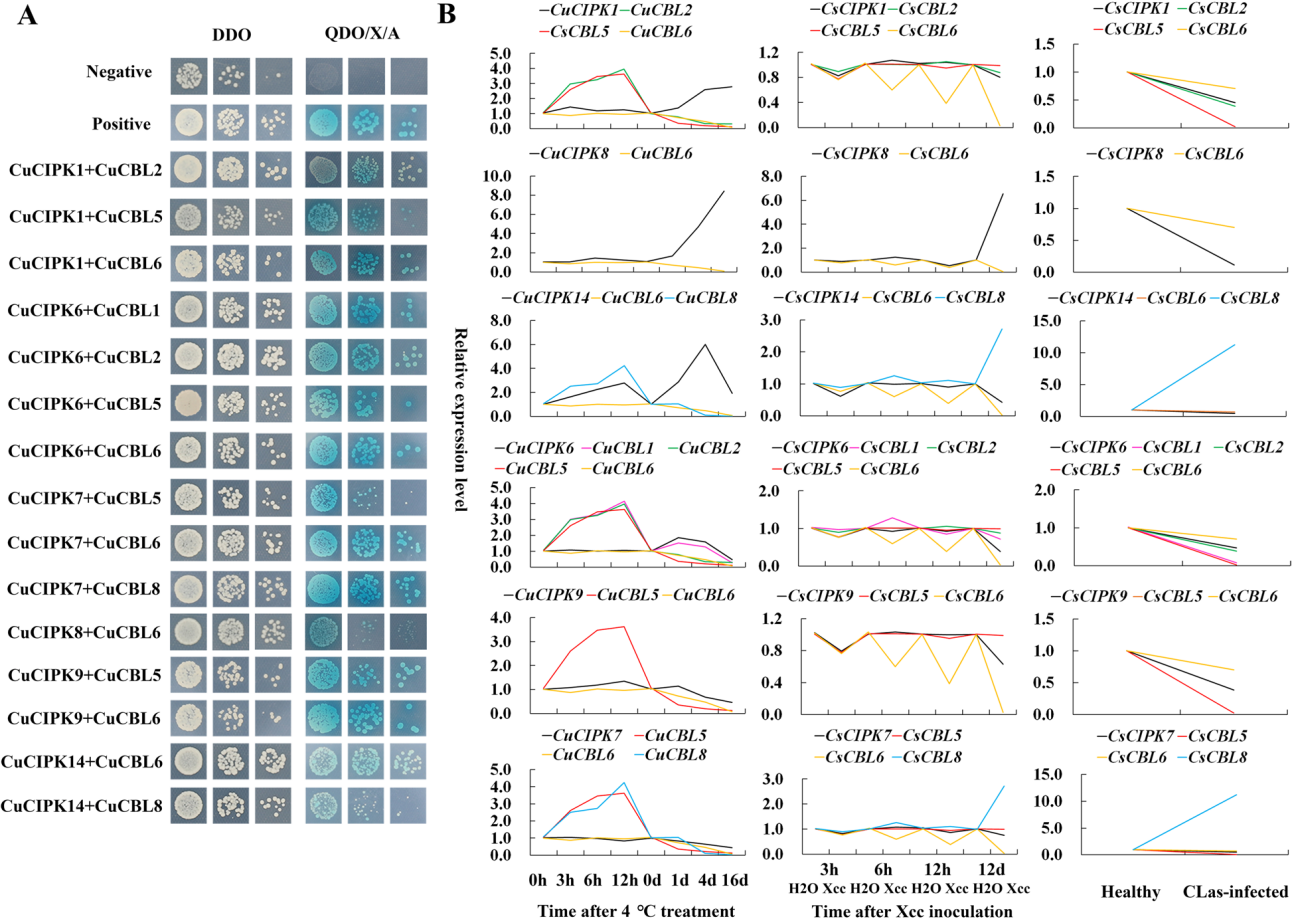
Interactive CBL-CIPK pairs in citrus verified by yeast two-hybrid assay (**A**) and comparison of their expression patterns under abiotic and biotic stresses (**B)**. A. DDO (SD/–Leu/–Trp) agar plates were used to select the positive mating clones, and QDO/X/A (SD/-Trp/-Leu/-His/-Ade/X-α-Gal/AbA) agar plates were used to select the positive interaction proteins. (pGADT7-T + pGBKT7-Lam) was used as the negative control, and (pGADT7-T + pGBKT7-53) was used as the positive control. B. The expression patterns are presented by line chart according to each *CIPK* and its interactive *CBLs*, and the same genes in different line charts are highlighted with the same colour

**Fig. 8:**
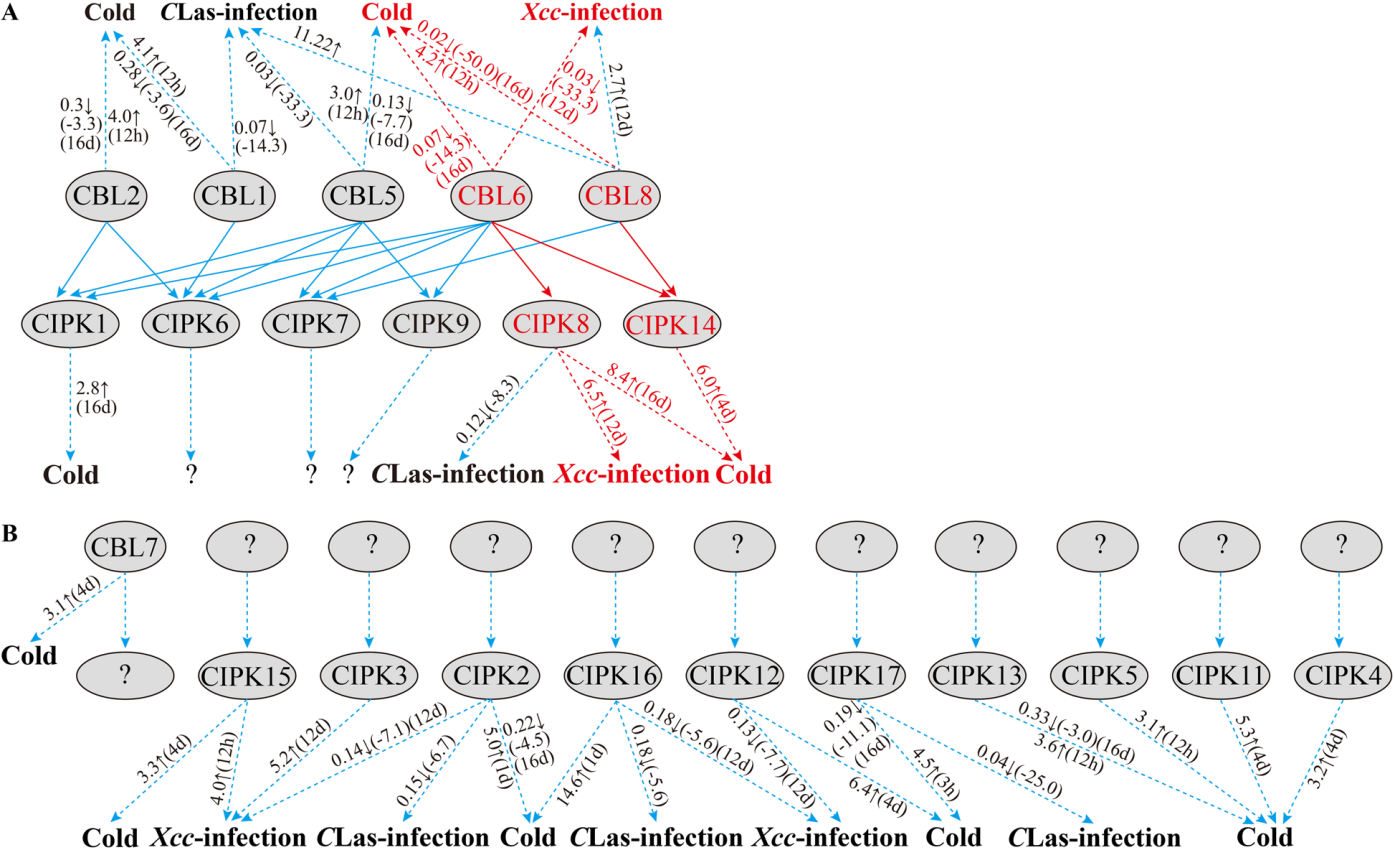
Potential CBL-CIPK signalling networks in citrus in response to abiotic and biotic stresses. A. Interaction relationships between CBL and CIPK proteins indicated by yeast two-hybrid assay and the observed gene expression responses to stresses according to qRT‒PCR assay. The predicted hub networks are highlighted by red arrows. B. The candidate key genes responsive to stresses according to expression analysis and the potential network need to be further investigated

To investigate how CBLs interact with CIPKs in citrus, we examined the interaction specificity of CBL and CIPK proteins by using yeast two-hybrid assay. Theoretically, eight CBLs and 17 CIPKs in citrus could form 136 possible interaction pairs. However, we found that only 15 CBL–CIPK pairs exhibited interactive relationships (Fig. 7A, Figure S3)*.* Interestingly, all recently evolved CBLs (except CBL4) had interacting CIPKs (accounting for 86.7% of the total interactive pairs) and were involved in multiple stresses (Fig. 8A). However, the majority of the candidate key stress-inducible CIPKs had no interacting CBLs (Fig. 8B). Among the positive interactive genes, *CBL1*, *5*, *6*, and *8* and *CIPK8* were substantially regulated by multiple stresses, including cold, *Xcc* infection and/or *C*Las infection, whereas *CBL2* and *CIPK1* and *14* were independently highly regulated by cold stress (Fig. 8A). *A*mong the negative interactive genes, *CIPK2*, *12*, *15*, *16*, and *17* were involved in multiple stresses, including cold, *Xcc* infection and/or *C*Las infection, whereas *CBL7* and *CIPK4*, *5*, *11* and *13* were independently regulated by cold stress, and *CIPK3* was uniquely responsive to *Xcc* infection (Fig. 8B). More importantly, CBL6 can interact with CIPK8 and respond to cold stress and *Xcc* infection in an antagonistic way, as both *CBL6* and *CIPK8* were highly regulated by the two stresses but showed opposite expression patterns (Fig. 7B and Fig. 8A). However, the CBL6/CBL8-CIPK14 interaction was independently involved in cold stress. Therefore, it can be inferred that CBL6-CIPK8 might be the common signalling network in response to cold and *Xcc* infection, while CBL6/CBL8-CIPK14 might be uniquely responsive to cold in citrus.

### Overexpression of *CuCIPK16* enhanced the cold tolerance of transgenic *Arabidopsis*

**Fig. 9:**
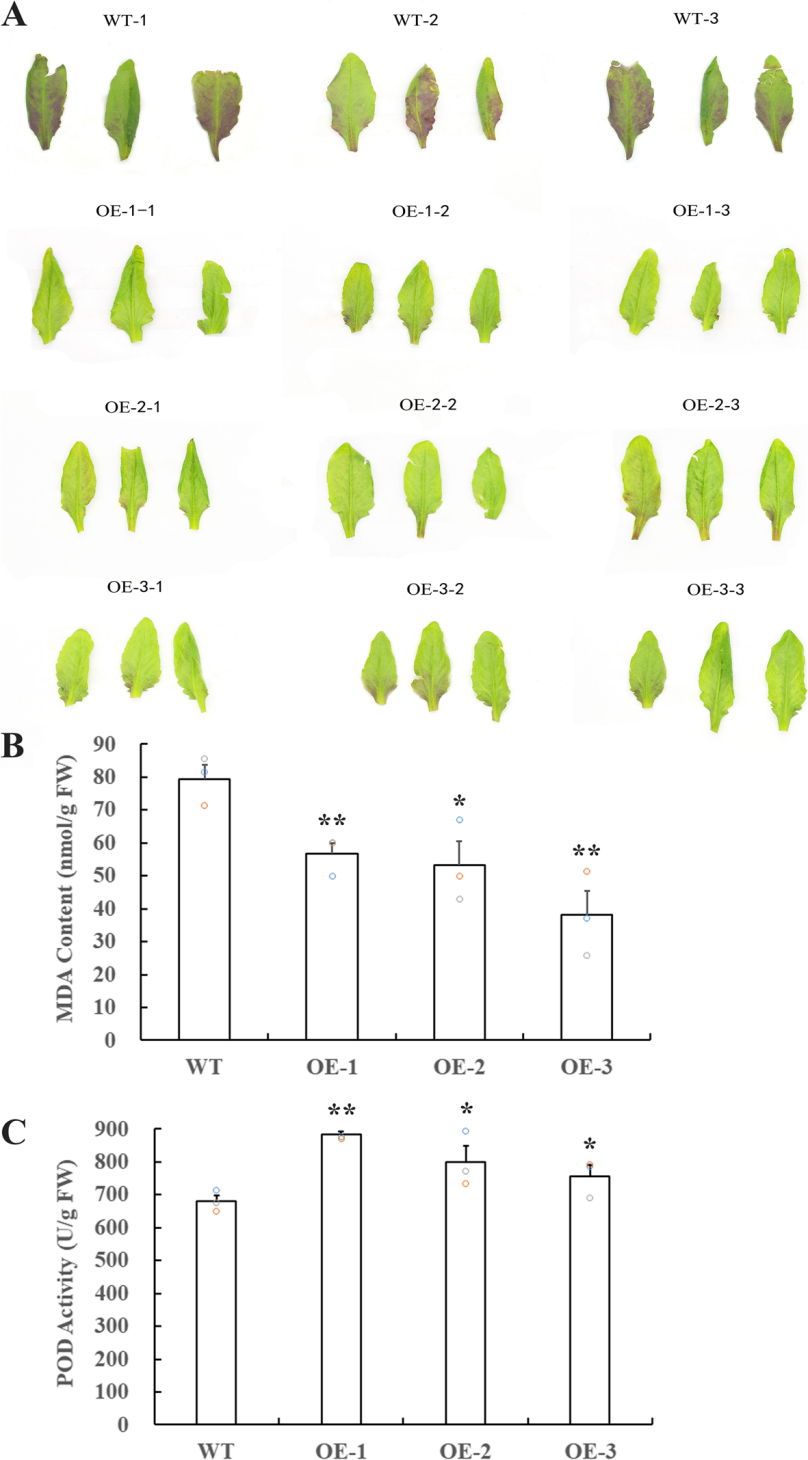
Comparison of phenotype (**A**), MDA content (**B**), and POD activity (**C**) between transgenic *Arabidopsis* lines and WT after cold treatment. For cold treatment, one-month-old *Arabidopsis* plants were subjected to 4 ℃ for seven days, followed by 0 ℃ treatment for one day and growth recovery at 21 ℃ for one day. Error bars in B and C indicate inferential statistics according to the SE. *indicates statistical significance (*p < 0.05; **p < 0.01; ***p < 0.001; Student’s t test) when compared to WT

According to the expression profiles, 75% of *CuCBL* genes and 71% of *CuCIPK* genes exhibited significant transcriptional changes upon cold stress, implying their important function in the cold response of ‘Guijing2501’ satsuma mandarin. Among the cold-responsive genes, the expression level of *CuCIPK16* was the highest under cold stress. Therefore, we cloned *CuCIPK16* and overexpressed it in *Arabidopsis* for functional analysis. Three transgenic *Arabidopsis* lines at the T2 generation were selected for the cold tolerance assay. One-month-old *Arabidopsis* plants were subjected to 4 ℃ for seven days, followed by 0 ℃ treatment for one day and growth recovery at 21 ℃ for one day. As a result, the abaxial leaf surface of the wild type (WT) turned deep purple on a large scale and exhibited severe freezing damage, whereas the three transgenic lines remained green or turned only light purple over a small range and exhibited slight freezing damage (Fig. 9A). Malondialdehyde (MDA) content and peroxidase (POD) activity were further measured. The results revealed that the transgenic plants had significantly improved POD activity but decreased MDA content relative to the WT (Fig. 9B-9C). These results suggest that overexpression of *CuCIPK16* enhanced the cold tolerance of transgenic *Arabidopsis* with higher POD activity and lower MDA content.

## Discussion

### The evolution of *CBLs* and *CIPKs* in citrus is closely associated with their adaptive feature for cold and bacterial infection

Conserved domain characteristics, intron/exon structures and duplications in gene families are often taken as imprints of gene evolution. In this study, the *CBL* and *CIPK* gene families in citrus can be divided into an ancient clade and a recent clade according to their conserved domain characteristics, which is in accordance with a previous analysis [35]. Moreover, *CIPKs* in the ancient clade are intron rich, whereas *CIPKs* in the recent clade are intron less. These characteristics of *CIPK* genes were also found in *Arabidopsis* [35], grape, apple, pineapple, and pear [28–31]. These results suggest that intron gain and loss are essential to the evolution of the *CIPK* family and that intron number has decreased during evolution. More importantly*,* CuCBL2 and CuCBL3, which are in the ancient clade, share the same amino acid sites in the conserved domains. However*,* the CBL proteins in the recent clade harbour more polymorphic amino acid sites. For instance, CuCBL6, which is tandemly duplicated from CuCBL5, harbours a shortened PFPF motif that is critical for CBL phosphorylation by CIPKs [1]. Additionally, CuCBL6 and CuCBL8 harbour the N-myristoylation site, which is critical for protein membrane binding [1]. Interestingly, these two CBL genes were further identified as signalling network hubs in response to cold stress and/or *Xcc* infection in citrus. Meanwhile*,* CBLs in the recent clade frequently interact with CIPKs and function in multiple stresses in citrus. In addition, the duplication events of citrus *CBLs* and *CIPKs* only exist in the recent clades, and the duplication might also contribute to the adaptive feature of citrus *CBLs* and *CIPKs* for cold and bacterial infection. Taken together, the evolution of *CBLs* and *CIPKs* in citrus might be closely associated with their adaptive feature for cold and bacterial infection.

### Specific expression patterns and stable transformation indicate the important roles of *CBLs* and *CIPKs* in the response to cold and bacterial infection in citrus

As the ubiquitous second messenger in plants, the increase in Ca^2+^ concentration is an early event during the perception of different types of stress signalling in plants [16, 36–39]. Ca^2+^ influx into the cytosol is also an early event during cold acclimatization, which can help plants increase freezing tolerance [36]. CBLs, one of the Ca^2+^ sensors, bind Ca^2+^ and interact with CIPKs to activate downstream targets that regulate specific biochemical processes and protect plants from stresses [16, 37, 38]. In tomato, the expression of *SlCIPK* genes was significantly upregulated in the early stages after 4 ℃ treatment [40]. In *Arabidopsis*, the expression of *AtCBL1* and *AtCIPK3, 7* was induced by cold, and disruption of *AtCIPK3* altered the expression patterns of many stress marker genes under cold stress [16, 41]. In addition, overexpression of the apple *MdCIPK6L* gene remarkably enhanced the tolerance of transgenic *Arabidopsis* and apple to cold stress [42]. In this study, 75% of *CuCBL* and 71% of *CuCIPK* genes in ‘Guijing2501’ satsuma mandarin were significantly regulated by cold acclimatization (4 ℃ treatment). Among these cold-responsive genes, 67% of *CuCBLs* and 83% of *CuCIPKs* were significantly upregulated starting in the early stages of cold treatment. Interestingly, the expression level of all four upregulated *CuCBLs* at early stages peaked at 12 h and then decreased. However, the majority of cold-responsive *CuCIPKs* were upregulated starting from early stages until later stages. The expression profiles of *CuCBLs* and *CuCIPKs* imply that CuCBLs, as Ca^2+^ sensors and CuCIPK activators, might function earlier than CuCIPKs, while CuCIPKs, as downstream target activators, might function until later stages. *CuCIPK16* was upregulated from early stages until later stages, and the peak expression level was the highest among the cold-responsive genes. Indeed, overexpression of *CuCIPK16* enhanced the cold tolerance of transgenic *Arabidopsis* with higher POD activity and lower MDA content. Taken together, these cold-responsive *CuCBL* and *CuCIPK* genes may be functionally involved in the cold response of ‘Guijing2501’ satsuma mandarin, and their potential function in citrus needs to be further studied.

The Ca^2+^ signalling pathway is also involved in the early steps of plant‒pathogen interactions [39]. CBLs and CIPKs can usually positively regulate disease resistance. In rice, OsCIPK14/15 is critical for the microbe-associated molecular pattern-induced defence signalling pathway [43], and CBL2-CIPK31-AKT1L was confirmed as a new signalling pathway to promote resistance to blast disease by increasing K^+^ uptake [17]. In wheat, the TaCBL4-TaCIPK5 complex positively modulates resistance to *Puccinia striiformis f.* sp. *tritici* depending on ROS [18]. In pepper, CaCIPK1 was found to mediate defence against *Phytophthora capsici* [44]. In cassava, MaCBL1/9-MaCIPK23 was revealed to enhance the defence response to *Xanthomonas axonopodis* pv. *Manihotis* [19]. However, AtCIPK6 negatively regulates effector- and PAMP-triggered immunity responses to *Pseudomonas syringae* in *Arabidopsis* [45]. In this study, except *CsCIPK15*, which was substantially upregulated at 12 h after *Xcc* inoculation, almost all the *CsCBL* and *CsCIPK* genes in sweet orange remained at a stable expression level at the early stages after *Xcc* inoculation. However, most of the *Xcc* infection-responsive genes (including *CsCIPK2*, *3*, *8*, *12*, and *16* and *CsCBL6* and *8*) were regulated at the later stage (12 d after *Xcc* inoculation). The possible reason might be the low *Xcc* inoculation concentration (10^4^ cfu/ml), leading to a relatively long time for *Xcc* proliferation in the leaf cells; thus, the typical infection symptoms appeared until 12 d after *Xcc* inoculation. In addition, the majority of the *C*Las infection-responsive genes (including *CsCIPK2*, *8*, *16*, and *17* and *CsCBL1* and *5*) were significantly downregulated, while only the *CsCBL8* gene was drastically upregulated. Whether these bacterial infection-responsive *CsCBL* and *CsCIPK* genes negatively or positively regulate the defence response of sweet orange still needs to be further studied.

### Citrus potential CBL-CIPK signalling networks involved in response to cold and bacterial infection in citrus

**Fig. 10:**
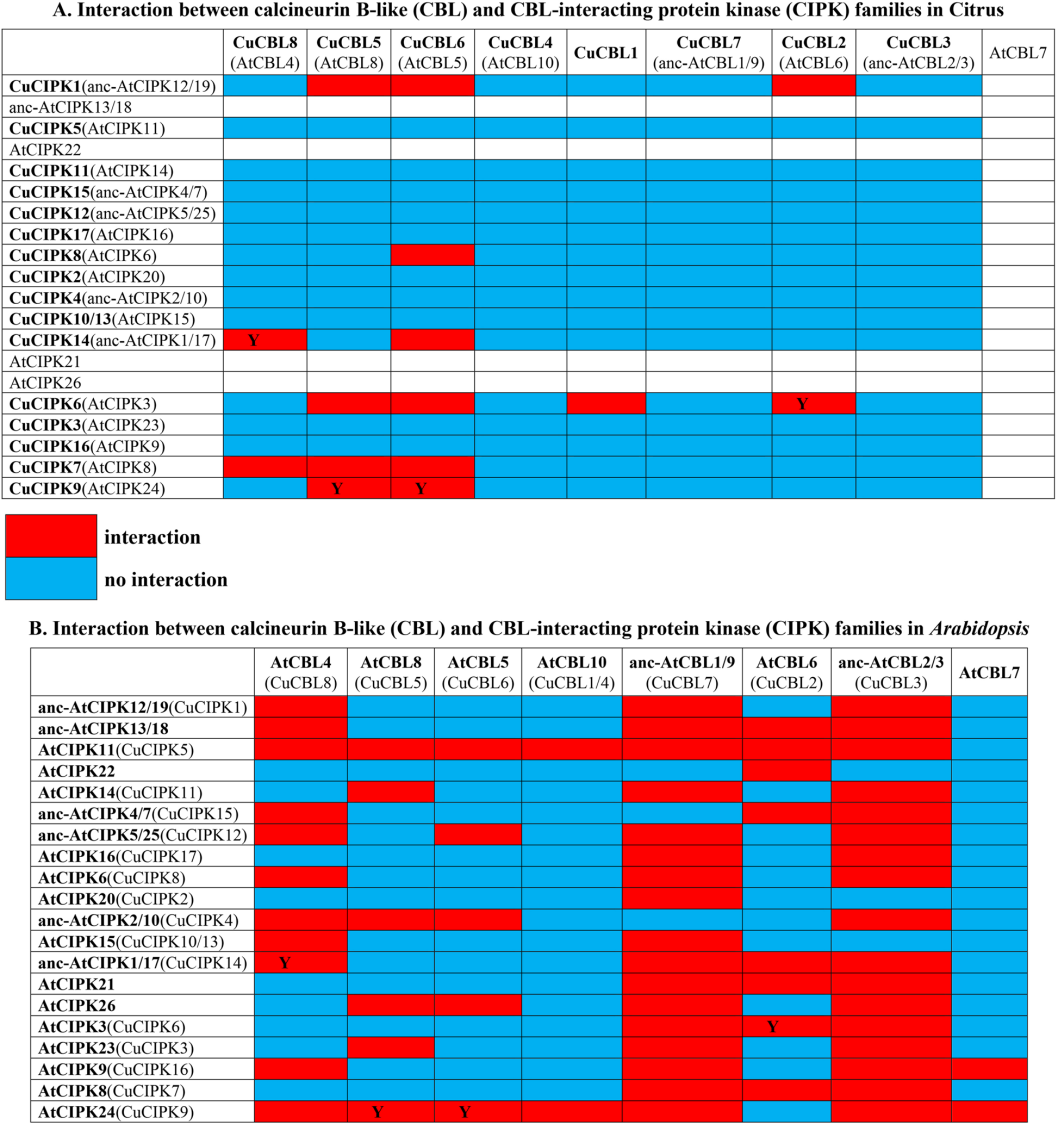
Comparison of the CBL-CIPK interaction patterns between citrus (**A**) and *Arabidopsis* (**B)**. The red square indicates interaction, and the blue square indicates no interaction for the test CBL-CIPK pairs. The white square indicates that there are no citrus orthologous genes corresponding to *Arabidopsis*, and the conserved CBL-CIPK pairs shared by citrus and *Arabidopsis* are marked with “Y” in the squares. The genes in the brackets are the corresponding orthologous genes. A. The CBL-CIPK interaction patterns for citrus were constructed according to the yeast two-hybrid assay in this study. B. The ancestral interaction states of CBLs and CIPKs before the α duplication event in *Arabidopsis* were reconstructed according to a previous study [35]. anc- indicates ancestral gene, and the gene pairs duplicated from the recent α event in *Arabidopsis* are considered as one ancestral gene

CBL-CIPK signalling networks have been found to decode intracellular calcium signals and regulate responses to various abiotic and biotic stresses in plants [1, 35]. In our study, CBL6-CIPK8 was identified as the common signalling network in response to cold and *Xcc* infection, while CBL6/CBL8-CIPK14 was uniquely responsive to cold in citrus. Compared with *Arabidopsis*, CBL-CIPK interactive networks might be different in citrus. For instance, in *Arabidopsis*, approximately 43% of CBL-CIPK showed interactive relationships, as indicated by a previous study [35], whereas only 11% of CBL-CIPK showed interactions in citrus (Fig. 10). Meanwhile, ancient anc-AtCBL1/9 and anc-AtCBL2/3 could interact with 85% of AtCIPKs in *Arabidopsis*; however, their orthologous proteins in citrus (CuCBL7 and CuCBL3) had no interactive CuCIPKs (Fig. 10). In contrast, the recently evolved CuCBL1, 5, 6, and 8 could form 13 interactive pairs (accounting for 86.7% of the total) with CuCIPKs (Fig. 10). Furthermore, Cs(u)CIPK8 may interact with Cs(u)CBL6 and is involved in the response to cold stress, *Xcc* infection (this study) and AMF colonization [33] in citrus*.* However, its orthologues in *Arabidopsis* (AtCIPK6) cannot interact with AtCBL5 (orthologue of Cs(u)CBL6) but interact with AtCBL4 (orthologue of Cs(u)CBL8) and are involved in the response to low K^+^, salt stress and bacterial infection [13, 45, 46]. Moreover, CuCBL6 can interact with CuCIPK14 and play a role in response to cold stress in citrus; however, their *Arabidopsis* orthologues cannot interact with each other [35]. The interaction between CuCBL8 and CuCIPK14 is conserved since their *Arabidopsis* orthologues (AtCBL4 and AtCIPK1) can also interact with each other. However, CuCBL8-CuCIPK14 is involved in the response to cold stress in citrus, while the *AtCBL4* gene is highly inducible by low K^+^ and salt stress, the expression of the *AtCIPK1* gene was not affected by these stress signals in *Arabidopsis* [13, 14, 47]. Therefore, CBL-CIPK signalling networks in response to abiotic and biotic stresses are extremely complicated, and the potential mechanism in citrus needs to be further studied.

## Conclusions

Cold is a major abiotic stress for citrus in regions prone to unpredictable winter weather, while Huanglongbing (HLB) and citrus canker disease are two devastating bacterial diseases that affect global citrus production. The Ca^2+^-CBL-CIPK signalling network plays important roles in the response to abiotic and biotic stresses in plants. How does this signalling network function in response to cold and infection by *C*Las or *Xcc* in citrus? Here, genome-wide range analyses based on evolution, gene expression and protein‒protein interactions were conducted to identify the key *CBLs*, *CIPKs* and CBL-CIPK interaction pairs implicated in responses to the above three stresses in citrus. As a result, *CBL1*, *5*, *6*, and *8* and *CIPK2*, *8*, *12*, *15*, *16*, and *17* in citrus were identified as common key genes responsive to multiple stresses, including cold, *Xcc* infection and/or *C*Las infection, whereas *CBL2*/*7*/*CIPK1/4/5/11*/*13*/*14* and *CIPK3* were unique key genes responsive to cold and *Xcc* infection, respectively. Meanwhile, CBL6-CIPK8 was the common signalling network in response to cold and *Xcc* infection, while CBL6/CBL8-CIPK14 was uniquely responsive to cold in citrus. In addition, stable transformation of *CuCIPK16* in *Arabidopsis* preliminarily verified its function in the cold response. The results from this comprehensive analysis may facilitate future functional characterization of individual citrus *CBLs* and *CIPKs* under specific stresses.

## Materials and methods

### Plant materials and stress treatment

The cold-tolerant satsuma mandarin (*C. unshiu*) variety ‘Guijing 2501’ was used as the material for cold treatment, and the *Xcc*-sensitive sweet orange (*C. sinensis*) variety ‘Taoye’ was used for *Xcc* inoculation. All plant materials employed for cold treatment and *Xcc* inoculation were kept at the Institute of Fruit and Tea, Hubei Academy of Agricultural Sciences, Wuhan, China. Healthy and uniform bud-grafting plants (2 years old) were grown in a greenhouse with a 16 h light/8 h dark photoperiod at 25 °C and routinely pruned to stimulate the growth of new leaves. For the cold acclimation assay, the ‘Guijing 2501’ satsuma mandarin plants were kept in a low-temperature (4 °C) growth chamber with a 16 h light/8 h dark photoperiod, and the leaves were sampled at certain time points (0 h/d, 3 h, 6 h, 12 h, 1 d, 4 d and 16 d). For the *Xcc* inoculation assay, fully expanded young leaves of ‘Taoye’ sweet orange were infiltrated with 10^4^ cfu/ml *Xcc* bacterial suspension, and sterile distilled water infiltration inoculation was performed as the control assay. The inoculated plants were kept in a high-humidity growth chamber with a 16 h light/8 h dark photoperiod and 85% humidity at 28 °C, and the leaves were sampled at certain time points (3 h, 6 h, 12 h and 12 d after *Xcc* inoculation). The symptoms were observed after 12 days of inoculation. The *C*Las-infected leaves with typical HLB symptoms from the 15-year-old ‘Yuanfeng’ navel orange trees were collected from Wugang County of Hunan Province, and healthy leaves in the same orchard were collected as the control samples. The main vein and petiole of the leaves were harvested for further *C*Las titre identification and expression analysis. All the samples were immediately frozen in liquid nitrogen and stored at –80 °C until further qRT‒PCR.

### Genome-wide identification and construction of phylogenetic trees for *CBL* and *CIPK* family genes in citrus

The reported CBL and CIPK protein sequences of *Arabidopsis* were downloaded from TAIR (http://www.Arabidopsis.org/) and then taken as queries to blast against *Citrus sinensis* V1.0 protein with identity > 90% and E-value < 1e–5 as cut-offs by the BLAST Tool in the citrus pangenome to breeding database (CPBD) (http://citrus.hzau.edu.cn/blast/query.php). The BLAST hits were further investigated and filtered manually based on the specific gene family motif features. EF-hand domains and PFPF motifs were used to validate the CsCBLs, and NAF/FISL motifs were used to validate the CsCIPKs. The genomic sequences of *CsCBLs* and *CsCIPKs* were employed to analyse the intron/exon structure by GSDS 2.0 [48]. The chromosomal locations of the *CsCBLs* and *CsCIPKs* were determined using TBtools software [49]. The *CuCBL* and *CuCIPK* genes with full-length CDSs were cloned by using cDNA from ‘Guijing 2501’ satsuma mandarin as the template. The protein sequences of the CuCBLs and CuCIPKs were deduced from the corresponding cloned nucleotide coding sequences, and the conserved domains/motifs were predicted by Pfam. The CBL and CIPK protein sequences of sweet orange, *Physcomitrella patens* and *Selaginella moellendorffii* were downloaded from CPBD (http://citrus.hzau.edu.cn) and PHYTOZOME v.11 (http://www.phytozome.net/), respectively. Multiple protein sequence alignments were then generated and trimmed using MEGA 7.0. A maximum likelihood phylogenetic tree was constructed based on the multiple sequence alignments, and 1000 bootstrap replicates were used to estimate the reliability of the tree topology by MEGA 7.0.

### Expression pattern analysis of *CBL* and *CIPK* genes in different sweet orange tissues and under different stresses

The RPKM values of *CsCBL* and *CsCIPK* genes in the sweet orange callus, leaf, flower, and fruit were retrieved from the RNA-seq data in sweet orange genome database (http://citrus.hzau.edu.cn/orange/). Samples collected from the above stress treatments were subjected to qRT‒PCR analysis. Total RNA was extracted using an RNAprep Pure Plant Kit (Tiangen, China) following the manufacturer’s instructions. RNA integrity was tested on 1.0% agarose gels stained with ethidium bromide, and the RNA concentration was calculated by a NanoPhotometer-NP80 (Implen, Germany). First strand cDNA was synthesized using the RevertAid™ First Strand cDNA Synthesis Kit (Fermentas, USA). qRT‒PCR was conducted using Applied Biosystems 7500 with PowerUP™ SYBR™ Green Master Mix (Thermo Scientific, USA). Specific primers for qRT‒PCR were designed by Primer Premier 5.0 software (Table S10). The specificity of the primers was further confirmed with a melting curve analysis after amplification of each tested gene. Each PCR pattern was verified using three biological replicates, and the experiment was repeated once. The *Cs(u)EF1α* gene was used as an internal reference gene to normalize the expression level. Samples before 4 °C treatment, infiltrated samples inoculated with sterile distilled water and samples confirmed without *C*Las infection were used as the internal controls for cold treatment, *Xcc* infection and *C*Las infection, respectively. The relative expression values were calculated by using the 2^−∆∆CT^ method. Standard error (SE) showing inferential statistics was calculated by the formula “STDEV.S/SQRT (number)” by using Excel software. Statistical analysis was performed using Student’s t test. A bar graph overlaying all the individual data points was employed to show the relative expression level. Error bars are indicated in the bar graph according to the SE. The asterisk indicates statistical significance according to Student’s t test (*p < 0.05; **p < 0.01; ***p < 0.001).

### Synteny analysis of the *CBL* and *CIPK* gene families

One Step MCScanX was conducted within the sweet orange genome using TBtools software [49]. E-values of 1e-10 and 5 of BlastHits were employed to filter the potential false gene duplication events. Advanced Circos in TBtools software was employed to display the synteny relationship of the orthologous *CBL* and *CIPK* genes within the sweet orange genome. The nucleotide coding sequences of *CsCBL* and *CsCIPK* genes were aligned using ClustalW 2.0, and then nonsynonymous substitution (Ka), synonymous substitution (Ks), and the Ka/Ks ratio were calculated using DNA Sequence Polymorphism v6.12.03 software.

### Yeast two-hybrid assay

The Matchmaker gold yeast two-hybrid System (Clontech, USA) was used to identify protein interactions. The eight *CuCBL* genes and 17 *CuCIPK* genes with full-length CDSs were cloned by specific primers (Table S11) and inserted into the pGADT7 vector and pGBKT7 vector, respectively. Then, the CuCBL-AD and CuCIPK-BD plasmids were transformed into the yeast strains Y187 and Y2HGold, respectively. After one-to-one yeast mating and spreading on DDO (SD/–Leu/–Trp) agar plates, the positive clones were examined by PCR. Subsequently, the positive clones were incubated in YPDA medium at 30 °C for 1 day. Ten microlitres of 1/1 (OD_600_ = 0.6), 1/10, and 1/100 dilutions were dripped on DDO (SD/-Trp/-Leu) agar plates and selective medium QDO/X/A (SD/-Trp/-Leu/-His/-Ade/X-α-Gal/AbA, supplemented with 8 mg/mL X-α-Gal and 125 ng/mL aureobasidin A) for 3 days to test protein interactions.

### Stable transformation of *CuCIPK16* in *Arabidopsis*

The full-length CDS of *CuCIPK16* was cloned using the primer CuCIPK16-OE-F/R and adapter primer attB1/attB2 (Table S11), inserted into the pDONR201 vector by Gateway BP Clonase II Enzyme Mix (Invitrogen, USA), and then ligated into the pK7YWG2 vector by Gateway LR Clonase II Enzyme Mix (Invitrogen, USA) following the manufacturer’s instructions. The constructs were verified by PCR and sequencing and then transformed into *Agrobacterium tumefaciens* strain GV3101. The floral dip method was employed for *Arabidopsis* transformation according to a previous study [50]. MS medium containing 50 μg ml^–1^ kanamycin was used to select the positive transformants, followed by genomic PCR and qRT‒PCR confirmation. Seeds of the transgenic lines were harvested until the T_2_ generation.

### Cold tolerance verification and measurement of MDA content and POD activity

Three transgenic *Arabidopsis* lines were selected for the cold tolerance assay. Seeds of three transgenic lines at the T_2_ generation and the wild type (WT) were germinated on soil pots and kept in a growth chamber with a 16 h light/8 h dark photoperiod at 21 °C. Two weeks later, three seedlings of each transgenic line and WT were independently transferred to each soil pot. For cold treatment, one-month-old *Arabidopsis* plants were subjected to 4 ℃ for seven days, followed by 0 ℃ treatment for one day and growth recovery at 21 ℃ for one day. The leaves were collected after cold treatment for MDA content and POD activity measurement. The MDA content and POD activity were determined using the relevant detection kits (A003-1–2 for MDA, A084-3–1 for POD, Nanjing Jiancheng Bioengineering Institute, China) following the manufacturer’s instructions.

## Supplementary Information

**Additional file 1: Figure S1.** Intron/exon structures and phylogenetic trees of the *CsCBL* and *CsCIPK* gene families.** Figure S2.** HLB-typical symptoms (**A**) and *C*Las titre quantification (**B**) for *C*Las-infected orange leaves and symptoms observed after 12 days of 10^4^ cfu/ml *Xcc* inoculation for *Xcc*-infected orange leaves (**C)**.** Figure S3.** 136 possible interaction sets for CuCBL and CuCIPK verified by yeast two-hybrid assay.** Table S1.** The full-length gene sequences of 8 *CsCBL* and 17 *CsCIPK* genes in sweet orange.** Table S2-S5.** Protein sequences of CBLs and CIPKs from sweet orange, ‘Guijing2501’ satsuma mandarin, *Arabidopsis*, *Physcomitrella patens* and *Selaginella moellendorffii*.** Table S6-S8.** Expression profiles of *Cs(u)CBL* and *Cs(u)CIPK* genes under cold stress, *C*Las infection and *Xcc* infection by qRT‒PCR.** Table S9.** One-to-one synteny relationships of the *CBL *or *CIPK* gene family within the sweet orange genome.** Table S10-S11.** Primer sequences used for qRT‒PCR, yeast two-hybrid assays and stable transformation.

**Additional file 2: File S1-S2.** Multiple alignment of amino acid sequences of CuCBLs and CuCIPKs from ‘Guijing2501’ satsuma mandarin.

## Data Availability

All data generated or analysed during this study are included in this published article and its supplementary information files.

## Abbreviations

CaM: Calmodulin
CML: Calmodulin-like protein
CBL: Calcineurin B-like protein
CDPK: Calcium-dependent protein kinase
CIPK: CBL-interacting protein kinase
PP2C: Protein phosphatase 2C
*C*Las: *Candidatus* liberibacter asiaticus
*Xcc*: *Xanthomonas citri* subsp. *citri*
HLB: Huanglongbing
Ka: Non-synonymous substitution
Ks: Synonymous substitution
Ka/Ks: Non-synonymous/synonymous substitution ratio
RPKM: Reads per kilobase per million mapped reads
WT: Wild type
OE: Over-expression
MDA: Malondialdehyde
POD: Peroxidase
