# Supplementary material for: Evolution, gene expression, and protein‒protein interaction analyses identify candidate CBL-CIPK signalling networks implicated in stress responses to cold and bacterial infection in citrus
Source: BMC Plant Biol. 2022 Sep 1;22:420. doi: 10.1186/s12870-022-03809-0 (PMC9434895; doi:10.1186/s12870-022-03809-0)
Supplement: Supplementary file 1 — Additional file 1: Figure S1. Intron/exon structures and phylogenetic trees of the CsCBL and CsCIPK gene families. Figure S2. HLB-typical symptoms (A) and CLas titre quantification (B) for CLas-infected orange leaves and symptoms observed after 12 days of 104 cfu/ml Xcc inoculation for Xcc-infected orange leaves (C). Figure S3. 136 possible interaction sets for CuCBL and CuCIPK verified by yeast two-hybrid assay. Table S1. The full-length gene sequences of 8 CsCBL and 17 CsCIPK genes in sweet orange. Table S2-S5. Protein sequences of CBLs and CIPKs from sweet orange, ‘Guijing2501’ satsuma mandarin, Arabidopsis, Physcomitrella patens and Selaginella moellendorffii. Table S6-S8. Expression profiles of Cs(u)CBL and Cs(u)CIPK genes under cold stress, CLas infection and Xcc infection by qRT‒PCR. Table S9. One-to-one synteny relationships of the CBL or CIPK gene family within the sweet orange genome. Table S10-S11. Primer sequences used for qRT‒PCR, yeast two-hybrid assays and stable transformation. [file 12870_2022_3809_MOESM1_ESM.zip › Supplementary Materials-20220804/File S1.pdf]

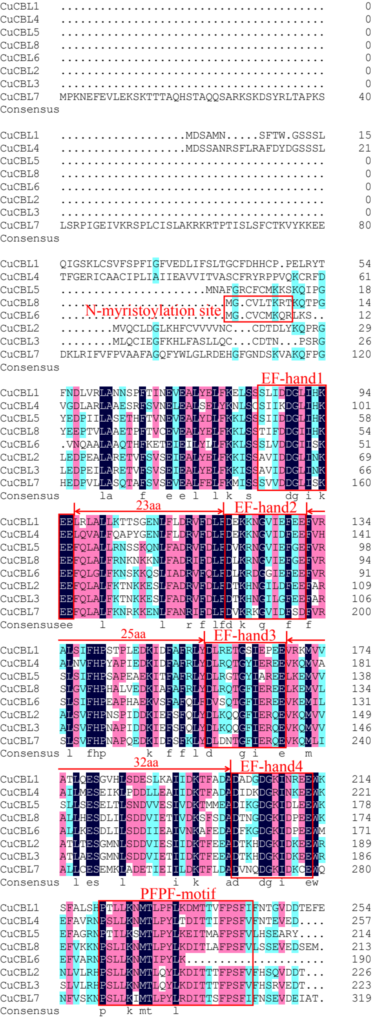
CuCBL1 0
CuCBL4 0
CuCBL5 0
CuCBL8 0
CuCBL6 0
CuCBL2 0
CuCBL3 0
CuCBL7 MPKNEFEVLEKSKTTTAQHSTAQQSARKSKDSYRLTAPKS 40
Consensus
CuCBL1 ...................MDSAMN.....SFTW.GSSSL 15
CuCBL4 ...................MDSSANRSFLRAFDYDGSSSL 21
CuCBL5 0
CuCBL8 0
CuCBL6 0
CuCBL2 0
CuCBL3 0
CuCBL7 LSRPIGEIVKRSPLCISLAKRKRTPTISLSFCTKVYKKEE 80
Consensus
CuCBL1 QIGSKLCSVFSPFIGFVEDLIFSLTGCFDHHCP.PELRYT 54
CuCBL4 TFGERICAACIPLIAIIEAVVITVASCFRYRPPVQKCRFD 61
CuCBL5 .......................MNAFGRCFCMKKSKQIPG 18
CuCBL8 ....MG.CVLTKRTKQTPG 14
CuCBL6 ...........................MG.CVCMKQRLKS.. 12
CuCBL2 ........MVQCLDGLKHFCVVVVNC...CDTDLYKQPRG 29
CuCBL3 ........MLQCIEGFKHLFASLLQC...CDTN...PSRG 26
CuCBL7 DKLRIFVFPVAAFAGQFYWLGLRDEHGFGNDSKVAKQFPG 120
Consensus
N-myristoylation site
EF-hand1
CuCBL1 FNDLVRLANNSPFTINEVEALYELFKKLSSSLIDDGLIHK 94
CuCBL4 VGDLARLAAESRFSVNELEALSELYKNLSCSIIKDGLIHK 101
CuCBL5 YEDPIILASETHFTVNEVEALYDLFKKLSSSIIDDGLIHK 58
CuCBL8 YEEPTVLAAETPFTVCEVEALYELFKKLSSTIFDDGIIHK 54
CuCBL6 .VNQAALAAQTHFKETEIEILYLLFKKLSSSLVDDGIISK 51
CuCBL2 LEDPEALARETVFSVSEIEALYELFKKISSAVIDDGLINK 69
CuCBL3 LEDPEILARETVFSVSEIEALYELFKKISSAVIDDGLINK 66
CuCBL7 HEDPVLLASQTAFSVSEVEALFELFKMISSSVVDDGLISK 160
Consensus la f e e l l k s dg i k
23aa
EF-hand2
CuCBL1 EELRLALLKTTSGENLFLDRVFDLFDEKKNGVIEFEEFVR 134
CuCBL4 EELQVALFQAPYGENLFLDRVFDLFDEKKNGVIDFEEFVH 141
CuCBL5 EEFQLALLRNSSKQNLFADRVFDLFDVKRNGVIEFGEFVR 98
CuCBL8 EEFQLALFRNKNKKNLFADRIFDLFDLKRNGVIEFGEFVR 94
CuCBL6 EEFQLGLFKNSKKQSLIADRVFQLFDLKRDGGIEFEEFVR 91
CuCBL2 EEFQLALFKTNKKESLFADRVFDLFDTKHNGILDFEEFAR 109
CuCBL3 EEFQLALFKTNKKESLFADRVFDLFDTKHNGILGFEEFAR 106
CuCBL7 EEFQLALFKNRKKENLFANRIFDLFDVKRKGVIDFSDFVR 200
Consensusee l l r f lfd k g f f
25aa
EF-hand3
CuCBL1 ALSIFHPSTPLEDKIDFAFRLYDLRETGSIEPEEVRKMVV 174
CuCBL4 ALNVFHPYAPIEDKIDFAFRLYDLRQTGYIEREEVKQMVA 181
CuCBL5 SLSIFHPSAPEAEKITFAFRLYDLRGTGYIAREELKEMVV 138
CuCBL8 SLGVFHPHALVEDKIAFAFRLYDLRQTGFIEREELKEMVL 134
CuCBL6 SLSIFHPEAPHAEKVSFAFQLFDVSQTGFIEREEVKEMIL 131
CuCBL2 ALSVFHPNSPIDDKIEFSFQLYDLKQQGFIERQEVKQMVV 149
CuCBL3 ALSVFHPNAPIDDKIEFSFQLYDLKQQGFIERQEVKQMVV 146
CuCBL7 SLSVFHPNAPQEDKIDFSFKLYDLDNTGFIERQEVKQMLI 240
Consensus l fhp k f f l d gi e m
32aa
EF-hand4
CuCBL1 ATLQESGVHLSDESLKAIIDKTFADADADGDGKINREEWK 214
CuCBL4 AILMESEIKLPDDLLEAIIDKTFADADIDKDGRINKEEWK 221
CuCBL5 SLLSESELTLSNDVVESIVDKTMMEADIKGDGKIDLEEWK 178
CuCBL8 ALLHESDLILSDDVIETIVDKSFSDADTNGDGKIDPEEWK 174
CuCBL6 ALLKESDLILSDDIIEAIVNKAFEDADFKGDGKIDPEEWM 171
CuCBL2 ATLTESGMNLSDDVIESIIDKTFEEADTKHDGKIDKEEWR 189
CuCBL3 ATLAESGMNLSDDVIETIIDKTFEEADTKHDGKIDKEEWR 186
CuCBL7 ALLCESEMKLADETIEIIIDKTFLDADVNQDGKIDKCEWQ 280
Consensus l es l i k ad dg i ew
PFPF-motif
CuCBL1 SFALSHPTLLKNMTLPFLKDMTTVFPSFIFNTGVDDTEFE 254
CuCBL4 EFAVRNPSLLKNMTLPYLTDITTIFPSFVFNTEVED.... 257
CuCBL5 EFAGRNPTILKSMTLPYLKEITMAFPSFVLHSEARY.... 214
CuCBL8 EFVKKNPSLIKNMTLPYLKDITLAFPSFVLSSEVEDSEM. 213
CuCBL6 EFVARNPSLLKNMTIPYLK..................... 190
CuCBL2 NLVLRHPSLLKNMTLQYLKDITTTFPSFVFHSQVDDT... 226
CuCBL3 SLVLRHPSLLKNMTLQYLKDITTTFPSFVFHSRVEDT... 223
CuCBL7 NFVSKNPSLLKIMTLPYLRDITTSFPSFIFNSEVDEIAT. 319
Consensus p k mt l
