# Supplementary figures and images for: Evolution, gene expression, and protein‒protein interaction analyses identify candidate CBL-CIPK signalling networks implicated in stress responses to cold and bacterial infection in citrus
Source: BMC Plant Biol. 2022 Sep 1;22:420. doi: 10.1186/s12870-022-03809-0 (PMC9434895; doi:10.1186/s12870-022-03809-0)

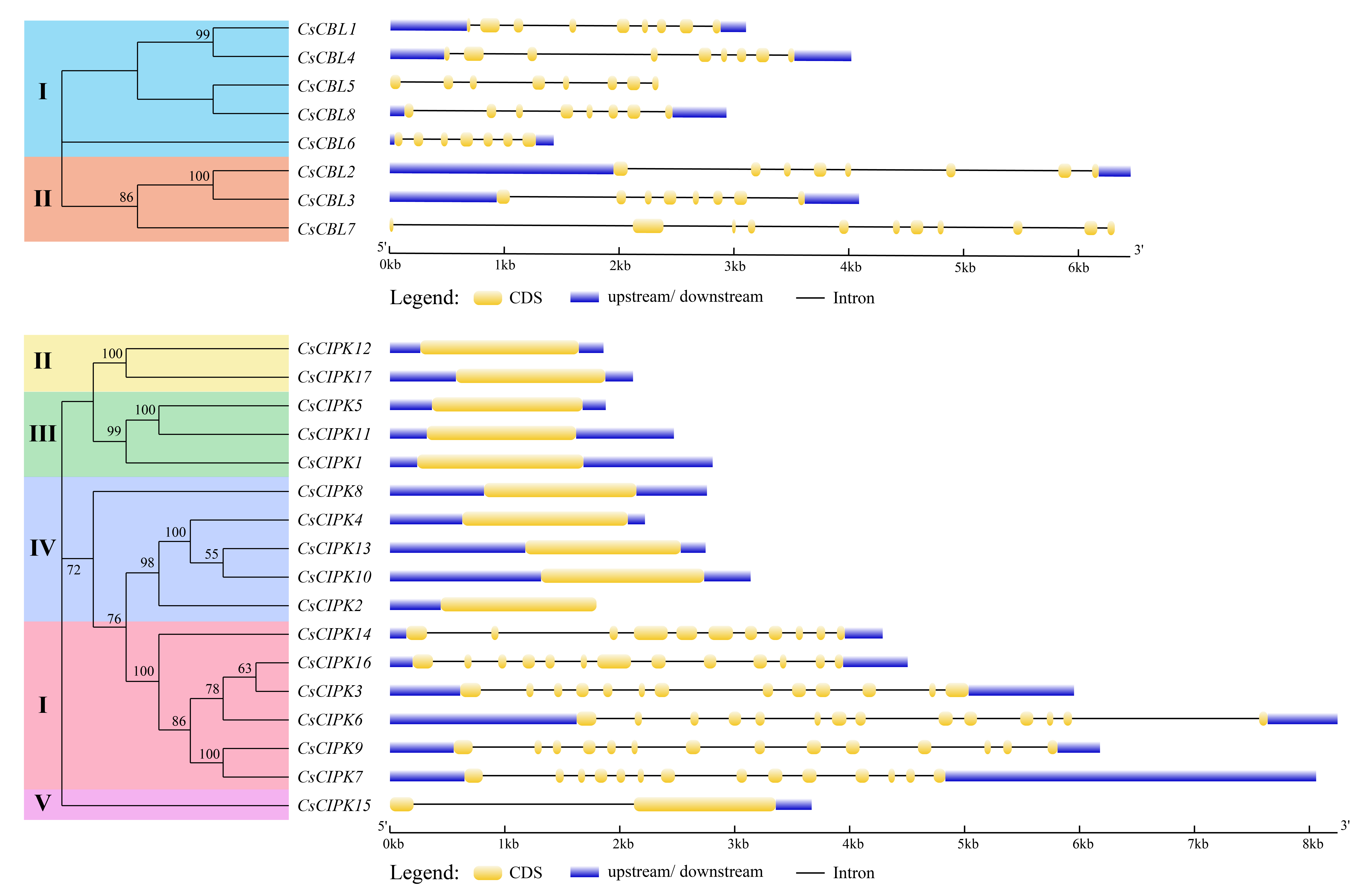

Supplement: Supplementary file 1 — Additional file 1: Figure S1. Intron/exon structures and phylogenetic trees of the CsCBL and CsCIPK gene families. Figure S2. HLB-typical symptoms (A) and CLas titre quantification (B) for CLas-infected orange leaves and symptoms observed after 12 days of 104 cfu/ml Xcc inoculation for Xcc-infected orange leaves (C). Figure S3. 136 possible interaction sets for CuCBL and CuCIPK verified by yeast two-hybrid assay. Table S1. The full-length gene sequences of 8 CsCBL and 17 CsCIPK genes in sweet orange. Table S2-S5. Protein sequences of CBLs and CIPKs from sweet orange, ‘Guijing2501’ satsuma mandarin, Arabidopsis, Physcomitrella patens and Selaginella moellendorffii. Table S6-S8. Expression profiles of Cs(u)CBL and Cs(u)CIPK genes under cold stress, CLas infection and Xcc infection by qRT‒PCR. Table S9. One-to-one synteny relationships of the CBL or CIPK gene family within the sweet orange genome. Table S10-S11. Primer sequences used for qRT‒PCR, yeast two-hybrid assays and stable transformation. [file 12870_2022_3809_MOESM1_ESM.zip › Supplementary Materials-20220804/Figure S1.jpg]

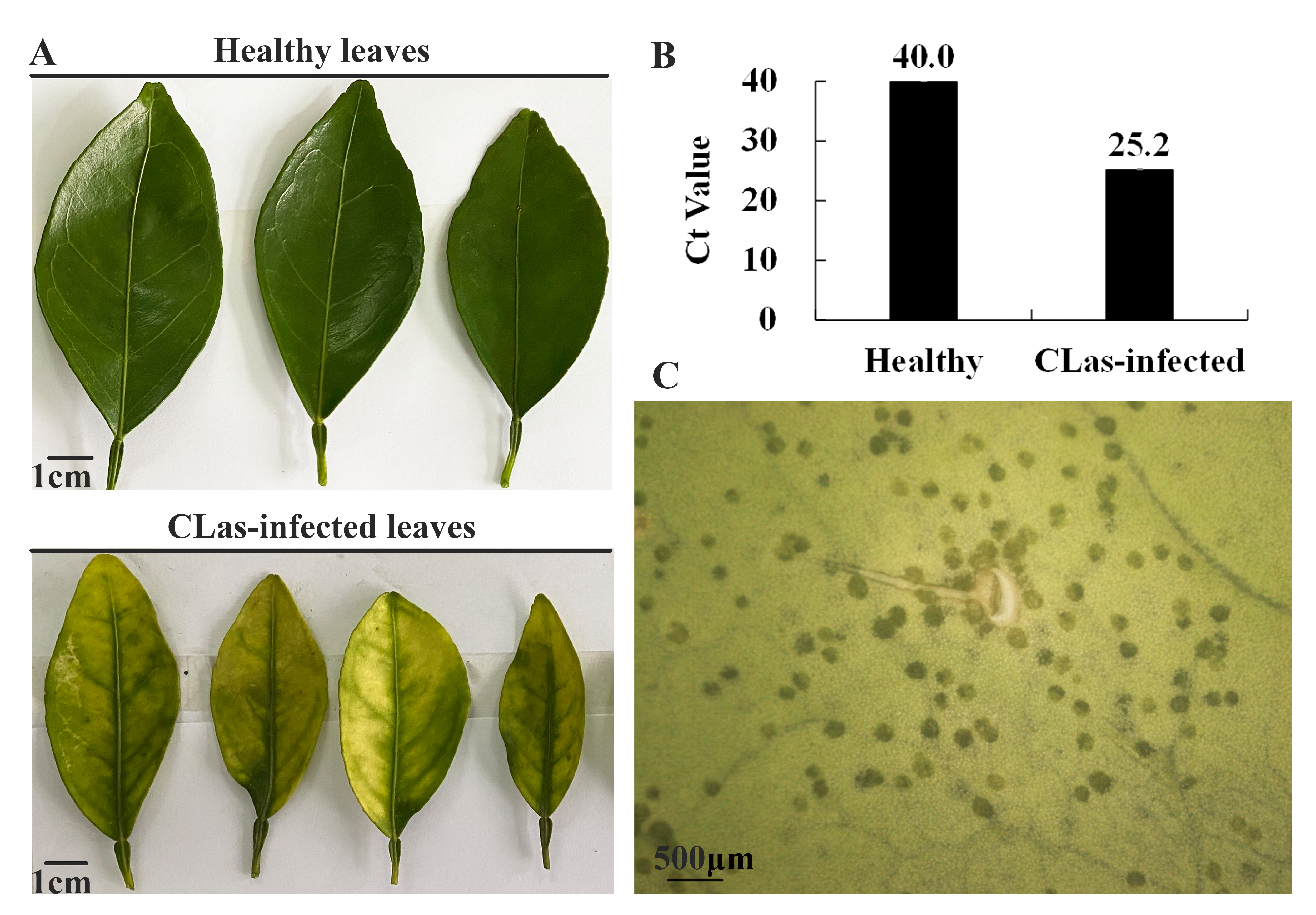

Supplement: Supplementary file 1 — Additional file 1: Figure S1. Intron/exon structures and phylogenetic trees of the CsCBL and CsCIPK gene families. Figure S2. HLB-typical symptoms (A) and CLas titre quantification (B) for CLas-infected orange leaves and symptoms observed after 12 days of 104 cfu/ml Xcc inoculation for Xcc-infected orange leaves (C). Figure S3. 136 possible interaction sets for CuCBL and CuCIPK verified by yeast two-hybrid assay. Table S1. The full-length gene sequences of 8 CsCBL and 17 CsCIPK genes in sweet orange. Table S2-S5. Protein sequences of CBLs and CIPKs from sweet orange, ‘Guijing2501’ satsuma mandarin, Arabidopsis, Physcomitrella patens and Selaginella moellendorffii. Table S6-S8. Expression profiles of Cs(u)CBL and Cs(u)CIPK genes under cold stress, CLas infection and Xcc infection by qRT‒PCR. Table S9. One-to-one synteny relationships of the CBL or CIPK gene family within the sweet orange genome. Table S10-S11. Primer sequences used for qRT‒PCR, yeast two-hybrid assays and stable transformation. [file 12870_2022_3809_MOESM1_ESM.zip › Supplementary Materials-20220804/Figure S2.jpg]

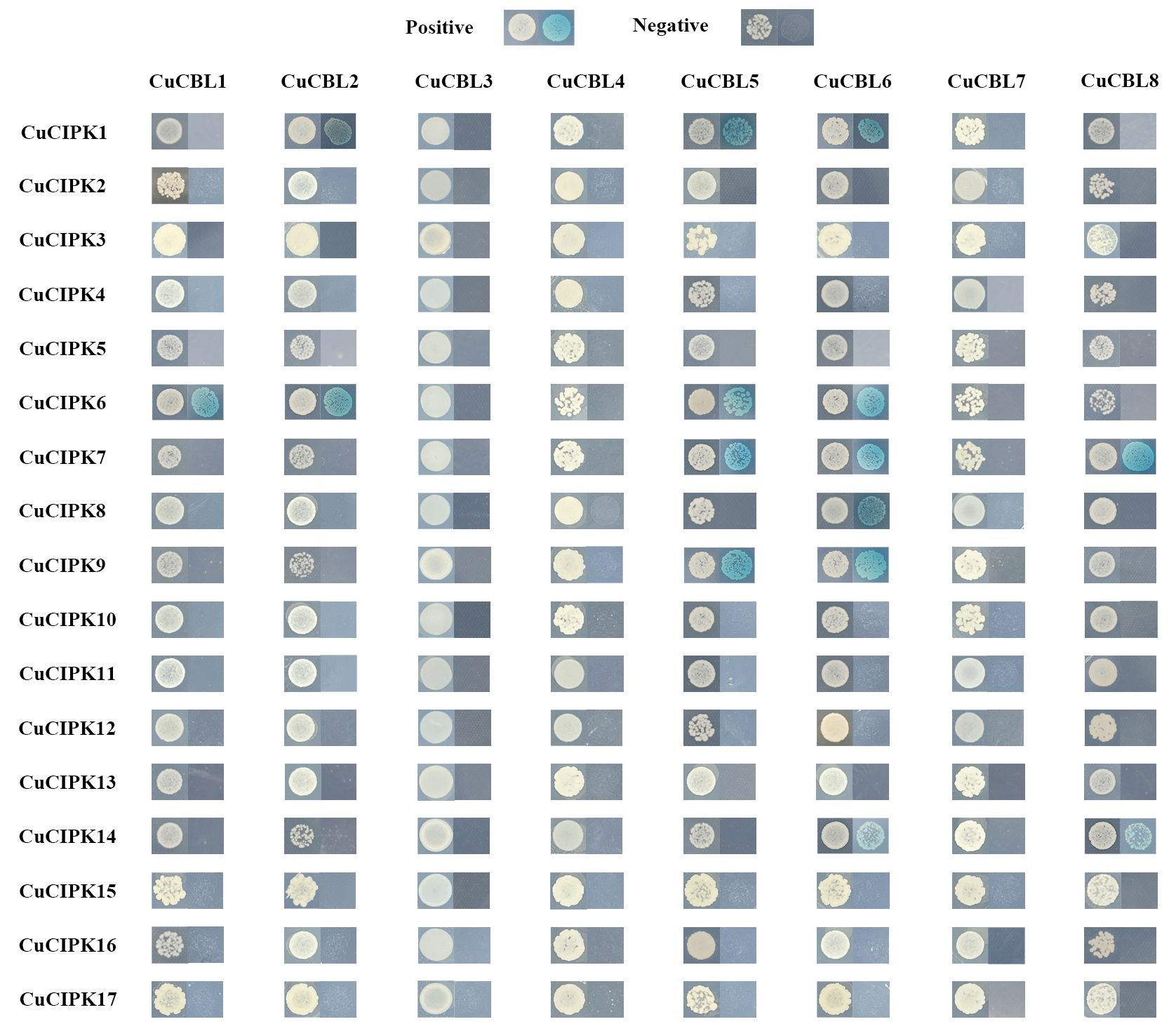

Supplement: Supplementary file 1 — Additional file 1: Figure S1. Intron/exon structures and phylogenetic trees of the CsCBL and CsCIPK gene families. Figure S2. HLB-typical symptoms (A) and CLas titre quantification (B) for CLas-infected orange leaves and symptoms observed after 12 days of 104 cfu/ml Xcc inoculation for Xcc-infected orange leaves (C). Figure S3. 136 possible interaction sets for CuCBL and CuCIPK verified by yeast two-hybrid assay. Table S1. The full-length gene sequences of 8 CsCBL and 17 CsCIPK genes in sweet orange. Table S2-S5. Protein sequences of CBLs and CIPKs from sweet orange, ‘Guijing2501’ satsuma mandarin, Arabidopsis, Physcomitrella patens and Selaginella moellendorffii. Table S6-S8. Expression profiles of Cs(u)CBL and Cs(u)CIPK genes under cold stress, CLas infection and Xcc infection by qRT‒PCR. Table S9. One-to-one synteny relationships of the CBL or CIPK gene family within the sweet orange genome. Table S10-S11. Primer sequences used for qRT‒PCR, yeast two-hybrid assays and stable transformation. [file 12870_2022_3809_MOESM1_ESM.zip › Supplementary Materials-20220804/Figure S3.jpg]
