# Supplementary material for: Evolution, gene expression, and protein‒protein interaction analyses identify candidate CBL-CIPK signalling networks implicated in stress responses to cold and bacterial infection in citrus
Source: BMC Plant Biol. 2022 Sep 1;22:420. doi: 10.1186/s12870-022-03809-0 (PMC9434895; doi:10.1186/s12870-022-03809-0)
Supplement: Supplementary file 2 — Additional file 2: File S1-S2. Multiple alignment of amino acid sequences of CuCBLs and CuCIPKs from ‘Guijing2501’ satsuma mandarin. [file 12870_2022_3809_MOESM2_ESM.doc]

**Supplementary materials**

Figure S1: Intron/exon structures and phylogenetic trees of the *CsCBL* and *CsCIPK* gene families.

Figure S2: HLB-typical symptoms (A) and *C*Las titre quantification (B) for *C*Las-infected orange leaves and symptoms observed after 12 days of 10^4^ cfu/ml *Xcc* inoculation for *Xcc*-infected orange leaves (C).

Figure S3: 136 possible interaction sets for CuCBL and CuCIPK verified by yeast two-hybrid assay.

Table S1: The full-length gene sequences of 8 *CsCBL* and 17 *CsCIPK* genes in sweet orange.

Table S2-S5: Protein sequences of CBLs and CIPKs from sweet orange, ‘Guijing2501’ satsuma mandarin, *Arabidopsis*, *Physcomitrella patens* and *Selaginella moellendorffii*.

Table S6-S8: Expression profiles of *Cs(u)CBL* and *Cs(u)CIPK* genes under cold stress, *C*Las infection and *Xcc* infection by qRT‒PCR.

Table S9: One-to-one synteny relationships of the *CBL* or *CIPK* gene family within the sweet orange genome.

Table S10-S11: Primer sequences used for qRT‒PCR, yeast two-hybrid assays and stable transformation.

File S1-S2: Multiple alignment of amino acid sequences of CuCBLs and CuCIPKs from ‘Guijing2501’ satsuma mandarin.
